# Vitamin levels in consumers: is diet, dissolved vitamins or vitamins produced by gut microbiota the most important source?

**DOI:** 10.1093/plankt/fbag078

**Published:** 2026-11-10

**Authors:** Samuel Hylander, Maciej J Ejsmond

**Affiliations:** Centre for Ecology and Evolution in Microbial model Systems (EEMiS), Department of biology and environmental science, Stuvaregatan 4, SE-392 31 Kalmar, Linnaeus University, Kalmar, Sweden; Institute of Environmental Sciences, Jagiellonian University, ul. Gronostajowa 7, PL-30-387 Kraków, Poland; Centre for Ecological and Evolutionary Synthesis, Department of Biosciences, P.O. Box 1066 Blindern, University of Oslo, NO-0316 Oslo, Norway

**Keywords:** zooplankton, vitamin, food web, thiamin, salmon, salmonid

## Abstract

Vitamins are continuously required for organismal metabolism. They are produced by phytoplankton, bacteria, and fungi and the general notion is that aquatic consumers, such as zooplankton and fish, obtain them from the diet. The focus here is on vitamin B_1_, called thiamine or thiamin, and the observation that concentrations of this vitamin decline with increasing trophic level. The mechanisms leading to this pattern are not well understood but we argue that it could be a sign of lower mass-specific metabolic demand for thiamine in larger compared to smaller consumers. Furthermore, we demonstrate the potential for alternative pathways of vitamin provision to consumers, apart from diet, including contributions from gut microbiota, or less likely, from the pool dissolved in water. How vitamins move through the food web as well as the metabolic demand of vitamins in differently sized consumers are essential factors to quantify to understand why top consumers, such as many salmonid species across the Northern Hemisphere, periodically develop thiamine deficiency, resulting in massive mortality.

## INTRODUCTION

Researchers recently found alarmingly high mortality in fry of several charismatic, economically and culturally important salmonid species in the Pacific Ocean (Reed *et al.,* 2023; Mantua *et al.,* 2025). It turned out that these fish lack a certain B vitamin, i.e. vitamin B_1_, also called thiamine or thiamin. Notably, these thiamine-related symptoms, had also been observed in several salmonids in the American Great Lakes and in the Baltic Sea (Bengtsson *et al.,* 1999; Fitzsimons *et al.,* 1999). Several other taxa including sea birds, alligators and seals might also be affected (Sepulveda *et al.,* 2004; Croft *et al.,* 2013; Balk *et al.,* 2016; Morner *et al.,* 2017). While we know that thiamine deficiency is the cause of unhealthy consumers the reasons why this vitamin sometimes becomes limiting are not well understood, although studies suggest that dietary components such as lipids and thiamine-degrading enzymes (thiaminases) could be involved (Harder *et al.,* 2018). Furthermore, in contrast to the well-developed literature on macronutrients (e.g. nitrogen and phosphorous), we do not know how this micronutrient travels through the food web and what other sources, other than diet, are significant (Fig. 1) (Kraft and Angert, 2017; Hylander *et al.,* 2024).

**Fig. 1:**
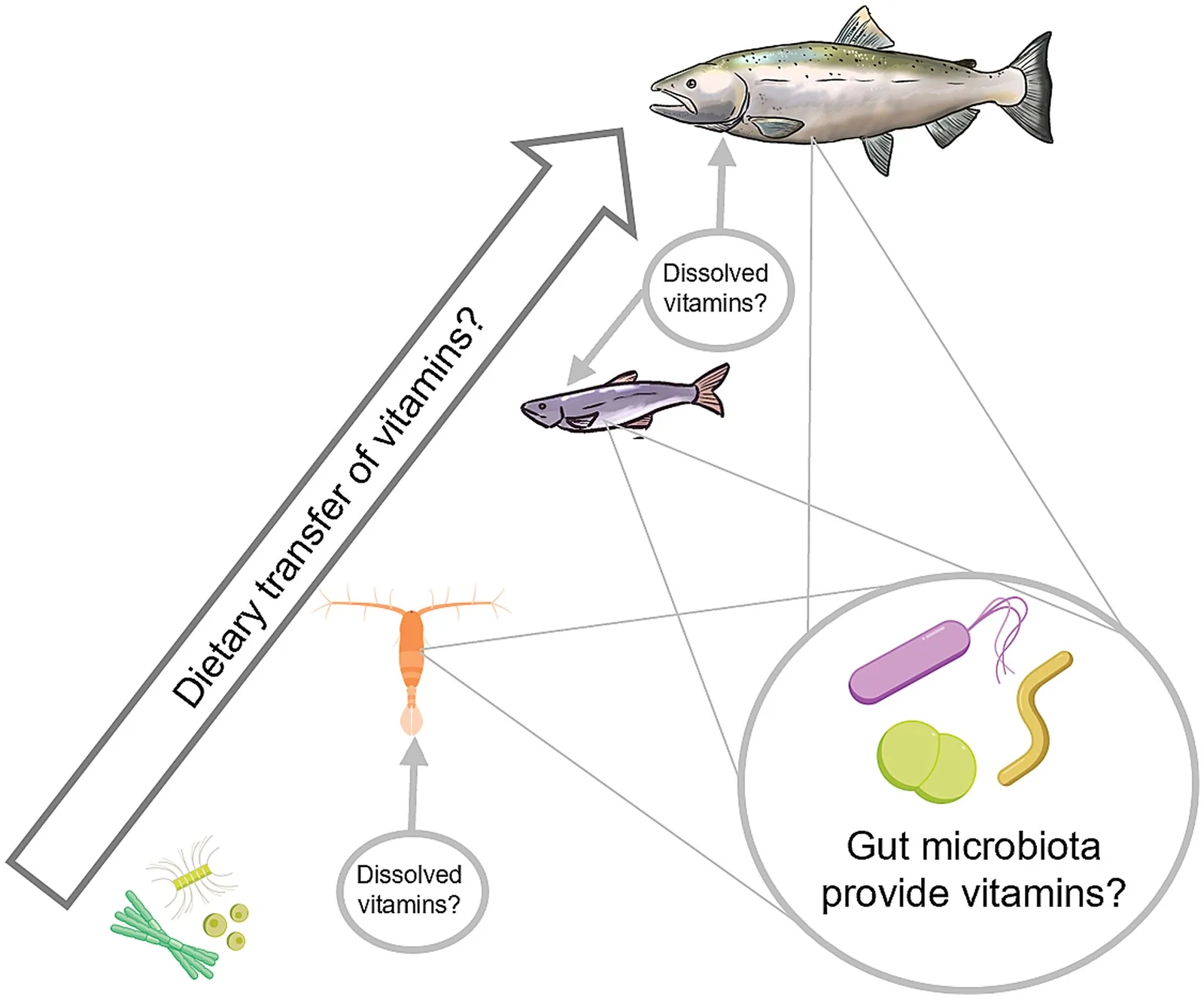
Vitamins, such as thiamine, are produced by primary producers such as phytoplankton and bacteria, and all consumers, such as zooplankton and fish, are dependent on this production. The question is how the vitamins reach the consumers, is it via dietary pathways, as generally assumed, or could dissolved vitamins or gut microbiota provide vitamins to the consumers? Redrawn from (Hylander *et al.,* 2024) (illustrations by Martin Brüsin and Marc Hauber).

Organisms need vitamins for a functioning metabolism (Manzetti *et al.,* 2014). The general assumption is that aquatic consumers, such as zooplankton and fish, receive vitamins from their diet. The primary question addressed here is whether this is a good assumption. Alternative sources of vitamins could be those dissolved in water, or deriving from gut microbes. Either of these sources would mean that vitamin levels in consumers are not directly linked to dietary sources, such as pelagic bacteria and phytoplankton (Fig. 1). Here we use the phytoplankton, zooplankton and thiamine dynamics as a model system to discuss the significance of dietary and other sources of vitamins for consumers in aquatic food webs (Fig. 1).

### Thiamine in aquatic food webs

Most of the early research on thiamine focused on dietary mechanisms in salmonids in search for the cause of the deficiency (Harder *et al.,* 2018). Only recently, more interest has been on the food web, and particularly, on the sources and how the vitamin reaches top consumers (Wienhausen *et al.,* 2022; Hylander *et al.,* 2024; Ejsmond *et al.,* 2025). Thiamine is produced by several taxa of phytoplankton, fungi, and bacteria with a range of strategies used to obtain the vitamin, including full synthesis, uptake of dissolved vitamin, and partial synthesis (Paerl *et al.,* 2018; Wienhausen *et al.,* 2022). Thiamine is water-soluble and degrades under certain abiotic conditions, e.g. under high pH, temperature and solar exposure (Carlucci *et al.,* 1969; Voelker *et al.,* 2021), which might be contributing to its scarcity. It has been assumed that all consumers, including zooplankton and fish, rely on ingestion of pelagic thiamine producers and only a few studies have attempted to understand how the vitamin is transferred within the food web (Ejsmond *et al.,* 2019; Wienhausen *et al.,* 2022; Hylander *et al.,* 2024). Published data on thiamine concentrations at different trophic levels illustrate that phytoplankton have highest concentrations and that there is a trophic dilution moving upwards in the food web (Fig. 2). Piscivorous fish only contain a small proportion of the phytoplankton concentration (Hylander *et al.,* 2024). This means that consumers operate at lower vitamin to carbon quotas compared to producers.

**Fig. 2:**
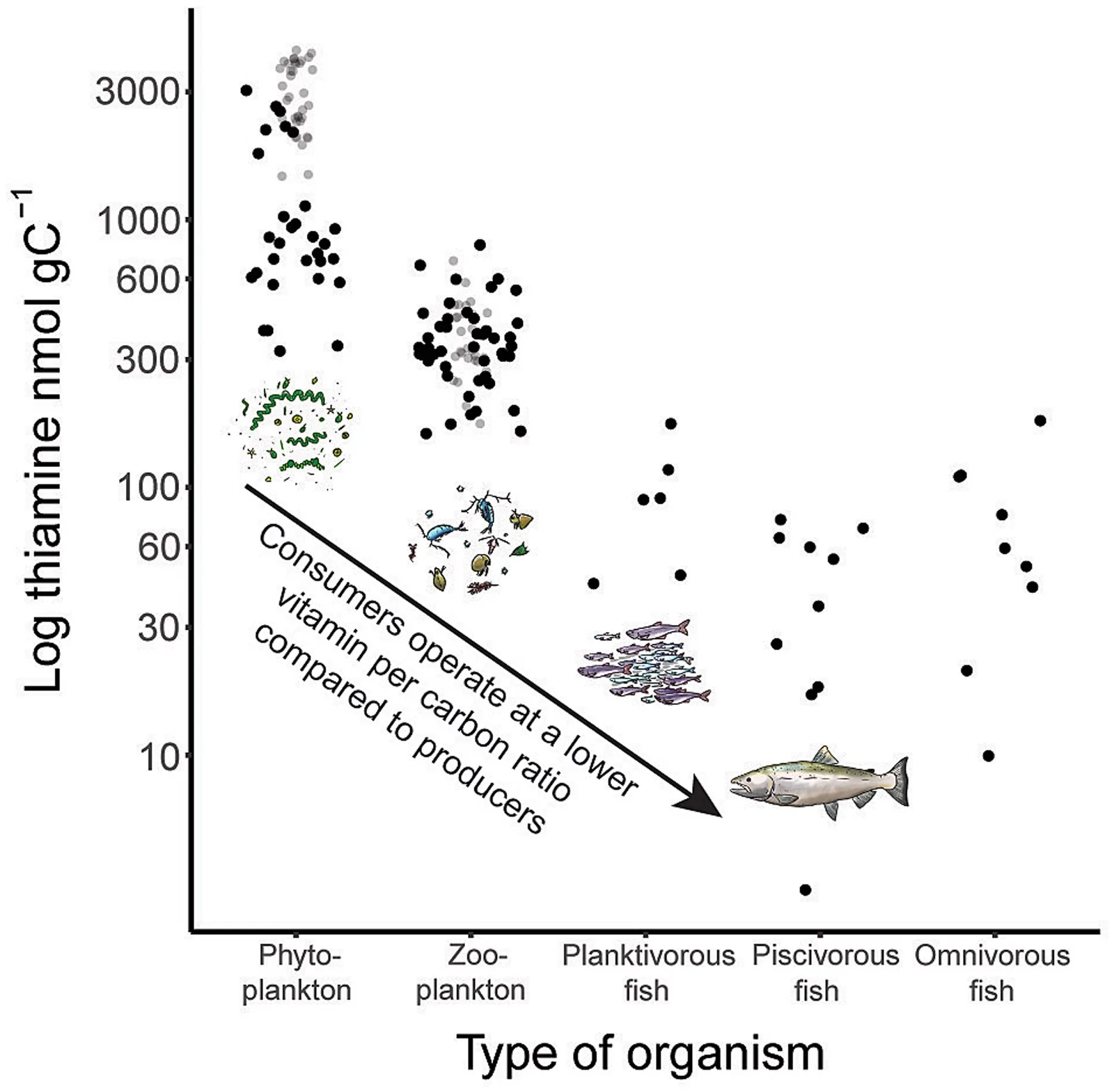
Literature compilation of thiamine concentrations normalized to carbon content in different trophic levels including phytoplankton, zooplankton, planktivorous fish, piscivorous fish, and omnivorous fish. Black dots represent literature data whereas grey dots are from mesocosm experiments. Redrawn from (Hylander *et al.,* 2024) (illustrations by Martin Brüsin).

The mechanisms leading to this trophic dilution are largely unknown. To begin to disentangle factors leading to trophic dilution, small scale laboratory experiments have been performed to study thiamine transfer from different phytoplankton diets to zooplankton. For example, zooplankton feeding on prey items rich in thiamine (e.g. cyanobacteria) have only moderately higher thiamine concentrations compared to zooplankton feeding on other prey items (Fridolfsson *et al.,* 2020; Fridolfsson *et al.,* 2023). Edibility of the prey item appears to be a good predictor for overall transfer of the vitamin. For example, zooplankton have higher thiamine concentrations compared to their prey when feeding on “palatable” phytoplankton, while concentrations are lower than in the prey when zooplankton are fed filamentous cyanobacteria (Fridolfsson *et al.,* 2020). Furthermore, if zooplankton thiamine concentrations are determined by phytoplankton thiamine concentrations, one would expect a positive correlation between their respective concentrations in field samples. However, such estimates only show a weak correlation (Fridolfsson *et al.,* 2019). Finally, outdoor mesocosm experiments with additions of large quantities of dissolved thiamine to the water, did not show any significant change in zooplankton thiamine concentrations (Hylander *et al.,* 2024).

Hence, the evidence that thiamine concentrations in zooplankton is determined by consumption of phytoplankton prey is relatively weak. This could potentially be due to constantly saturating levels of thiamine in prey items, or that zooplankton maintain thiamine homeostasis, as has been shown for other nutrients (Andersen and Hessen, 1991). Threshold concentrations required in the diet to sustain sufficient thiamine levels has been identified for larger organisms, such as fish (Woodward, 1994; Kamalam *et al.,* 2020), but is not well known in zooplankton. Hence, more studies are needed to understand the mechanisms underlying trophic dilution of thiamine. Future studies should apply mass-balance approaches that quantify dietary and dissolved uptake in consumers, metabolic processes and the influence of vitamin to macronutrient stoichiometry on trophic transfer efficiency across trophic levels, as has been done for other nutrients and elements (Reinfelder *et al.,* 1998; Mehner *et al.,* 2022).

Future approaches should also consider the allometric relationship between metabolic rate and body size. Typically, body size increases by orders of magnitude along the aquatic food web with mass-specific rates such as metabolic and growth rates decreasing with organism size (Makarieva *et al.,* 2008; Kiorboe and Hirst, 2014). This might explain the lower thiamine concentrations in larger compared to smaller organisms in the food web. However, other mechanisms leading to vitamin loss or degradation in each trophic transfer also need further consideration.

### Microbial provision of thiamine

Moving beyond the dietary sources of thiamine, other potential routes for thiamine provision could result from dissolved thiamine in the water or from the gut microbiota. While dissolved thiamine does occur in water, it is at picomolar concentrations (Sanudo-Wilhelmy *et al.,* 2012), questioning the importance of this source for consumers. In terms of the gut microbiota, terrestrial herbivores and omnivores rely heavily on microbial provision of vitamins. For example, ruminants and humans can base a large proportion of their needs on microbially produced vitamins (Breves *et al.,* 2009; Nysten and Van Dijck, 2023). Insects can similarly receive thiamine from the gut microbiota (Sannino *et al.,* 2018). Experiments with *Drosophila* demonstrate that this taxon can rely solely on thiamine from the gut microbiota without any dietary source (Sannino *et al.,* 2018). It is well known that zooplankton have a functionally important gut microbiome, including functions such as digestive help, detoxification and vitamin provision (Harris, 1993; Gorokhova *et al.,* 2020; Calbet, 2026). The magnitude of vitamin provision in zooplankton is not well known generally. However, copepod-associated microbiomes contain bacteria with capacity to produce B-vitamins (Sadaiappan *et al.,* 2021; Calbet, 2026).

To evaluate whether external thiamine, either dissolved in the water column or excreted from GI-tract microbes, could be of importance for aquatic consumers (e.g. zooplankton and fish) we estimated the theoretical daily uptake from water surrounding the consumer or from digestive fluids using a combination of published thiamine and riboflavin kinetics data (Fig. 3; see Supplementary material). Vitamins in these digestive fluids could come from dietary sources or from gut microbes. We also varied organ sizes for uptake, e.g. intestine or gills (see Supplementary material for modeling approach). Dissolved thiamine concentrations in nature generally range from non-detectable to ~1 nM (Sanudo-Wilhelmy *et al.,* 2012; Suffridge *et al.,* 2024). Hence, this back-of-an-envelope calculation (Fig. 3) indicates that observed picomolar water concentrations cannot fully support fish thiamine demands, and only partly support zooplankton demands (gray areas; Fig. 3). This is assuming that a large proportion of the zooplankton body can be used for vitamin uptake—which is not known (Fig. 3). Hence, for fish, uptake via gills from the dissolved thiamine pool seems unlikely to have any major effect on organismal thiamine status. It is also not known whether gills have any ability to take up dissolved vitamins at all. When concentrations are in the nM range, we can expect physiologically important uptake in consumers, i.e. from dietary or microbially derived sources of vitamins (Fig. 3). Hence, gut microbial production of thiamine, if available for the host, could be important for both zooplankton and fish. However, it remains to be determined to what extent this mechanism is occurring in zooplankton gut microbiomes. Fish, such as salmonids, spend long periods (months) in starvation before reproduction. One can hypothesize that microbial thiamine provision in the GI-tract might be of importance to the fish when dietary sources are absent, as has been demonstrated in *Drosophila* (Sannino *et al.,* 2018).

**Fig. 3:**
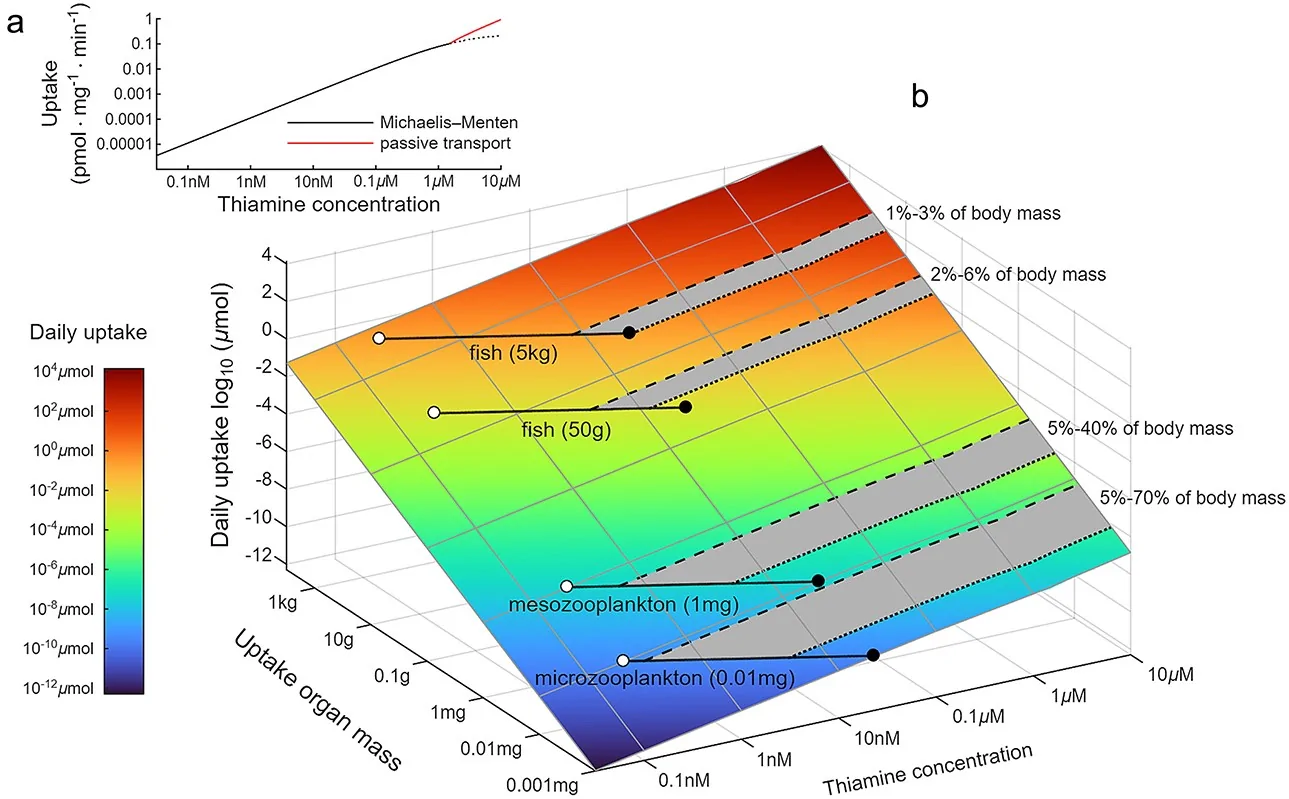
The rate of thiamine uptake determined by the size of the uptake organ and the external thiamine concentration. (a) The thiamine uptake rate, per mg of uptake organ, was calculated assuming Michaelis–Menten kinetics for concentrations of up to 1.5 μM, and by passive transport for concentrations above 1.5 μM. (b) Daily thiamine uptake rate was determined by the mass of the uptake organ and the external thiamine concentration. The black solid lines represent isolines with the total daily thiamine requirement of an organisms with a given body mass (see the labels at the lines). The white dots at the end of the lines correspond to the mass of the uptake organ being equal to the total body mass while black dots match organs at 1% of body mass. For simplicity, we assumed that the mass-specific daily requirement does not differ between organisms of different sizes and equals to 10 μg/day/kg, calculated from 1 mg/kg diet requirement in rainbow trout with feed ration at 1% of body weight (Kamalam *et al.,* 2020). In future, more detailed approaches could also test requirements that are higher for zooplankton than for fish due to allometry of metabolic rate (now not available in this model). Grey areas correspond to combinations of external concentration and uptake organ size that allow the daily requirements to be met through uptake. The gray areas are demarcated by dashed and dotted lines, which set the minimum and maximum size of the organ relative to body size (see the labels to the right of the gray areas for the assumed tested range of organ size).

## CONCLUSIONS

Based on our review of the current knowledge, we conclude that the key assumption that vitamins in aquatic consumers reach higher trophic levels only through dietary sources lacks support. In addition to diet, zooplankton, and potentially other consumers such as fish, might benefit from vitamins produced by gut microbes as a route of vitamin provision. Furthermore, a disruption of gut microbiota could potentially lead to thiamine deficiency in consumers. Therefore, we recommend that future studies should combine traditional dietary assessment and allometric metabolic approaches with studies quantifying the importance of these other routes whereby consumers, such as zooplankton and fish, receive their metabolically critical vitamins.

## Supplementary Material

Supplementary_material_Hyalnder_Ejsmond_ME_1_fbag078

## Data Availability

This manuscript does not provide any original data but instead synthesizes and discusses results from previously published studies. Relevant citations are provided in conjunction to figures 2 and 3.
