# Supplementary material for: Vitamin levels in consumers: is diet, dissolved vitamins or vitamins produced by gut microbiota the most important source?
Source: J Plankton Res. 2026 Nov 10;48(6):fbag078. doi: 10.1093/plankt/fbag078 (PMC13750371; doi:10.1093/plankt/fbag078)
Supplement: Supplementary file 1 [file supplementary_material_hyalnder_ejsmond_me_1_fbag078.docx]

**Supplementary material for:**

**Vitamin levels in consumers: Is diet, dissolved vitamins or vitamins produced by gut microbiota the most important source?**

Samuel Hylander^1^, Maciej J. Ejsmond^2^

1. Corresponding author, Centre for Ecology and Evolution in Microbial model Systems (EEMiS), Linnaeus University, Sweden
2. Maciej J. Ejsmond; Institute of Environmental Sciences, Jagiellonian University, Poland;
   Centre for Ecological and Evolutionary Synthesis, Department of Biosciences, University of Oslo, Norway.

**Calculation of the daily thiamine uptake rate**

To calculate the rates of thiamine uptake in consumers, we used data on thiamine duodenal absorption at high concentrations in rats (Hoyumpa *et al.*, 1975) and the Michaelis–Menten kinetics of riboflavin uptake at low concentrations in salmon (Casirola & Ferraris, 1997). We used the Michaelis–Menten equation parameters for riboflavin (vitamin B₂) from Casirola and Ferraris (1997) because both thiamine and riboflavin are water-soluble and their physiological uptake is governed by vitamin-specific sodium-independent, pH-dependent carrier-mediated mechanisms. After converting the uptake rates reported by Hoyumpa et al. (1975) and Casirola and Ferraris (1997), the uptake were comparable between rats and rainbow trout in terms of levels and saturation.
We assumed that the thiamine uptake rate for concentrations below 1.5 µM was determined by the Michaelis–Menten equation (see (Casirola & Ferraris, 1997)), while for concentrations above 1.5 µM, thiamine uptake was driven by passive processes proportional to the external concentration (see (Hoyumpa *et al.*, 1975)). While Hoyumpa et al. (1975) reported a thiamine uptake rate of 4 ng/5 min, per cm of duodenum, at a concentration of 1.5 µM, we assumed this was equivalent to an uptake rate of 0.1021 pmol/min, per mg of uptake tissue, at a concentration of 1.5 µM, as calculated from Casirola and Ferraris (1997) for riboflavin. We also calculated that a thiamine uptake rate of 65 ng/cm/5 min at a concentration of 17.5 µM (from (Hoyumpa *et al.*, 1975)) is equivalent to a rate of 1.6591 pmol/mg/min at an external concentration of 17.5 µM.
 Overall, in our model the actual thiamine uptake rate *U* (pmol/mg/min) was given by

Eq 1. $\left\{ \begin{aligned} U=\frac{U_{max}C}{K_{m}+C} for C\leq1.5 \mu mol \\ U=0.0973C-0.0439 for C>1.5 \mu mol \end{aligned} \right.$
where *U*_max_ and *K*_m_ are maximum uptake rate and Michaelis constant for riboflavin, with values of *U*_max_= 0.26 pmol/mg/min and *K*_m_= 2.32 µM (from (Casirola & Ferraris, 1997)), and *C* matches the external concentration in µM.

References

Casirola, D. M. and Ferraris, R. P. (1997) Intestinal absorption of water-soluble vitamins in rainbow trout (Onchorhynchus mykiss). *Comp Biochem Physiol A Physiol,* **116,** 273–9.

Hoyumpa, A. M., Jr., Middleton, H. M., 3rd, Wilson, F. A. and Schenker, S. (1975) Thiamine transport across the rat intestine. I. Normal characteristics. *Gastroenterology,* **68,** 1218–27.
